# A Simple Lattice Model That Captures Protein Folding, Aggregation and Amyloid Formation

**DOI:** 10.1371/journal.pone.0085185

**Published:** 2014-01-15

**Authors:** Sanne Abeln, Michele Vendruscolo, Christopher M. Dobson, Daan Frenkel

**Affiliations:** 1 IBIVU - Deptartment of Computer Science, VU University, Amsterdam, The Netherlands; 2 Deptartment of Chemistry, University of Cambridge, Cambridge, United Kingdom; University of Maryland School of Medicine, United States of America

## Abstract

The ability of many proteins to convert from their functional soluble state to amyloid fibrils can be attributed to inter-molecular beta strand formation. Such amyloid formation is associated with neurodegenerative disorders like Alzheimer's and Parkinson's. Molecular modelling can play a key role in providing insight into the factors that make proteins prone to fibril formation. However, fully atomistic models are computationally too expensive to capture the length and time scales associated with fibril formation. As the ability to form fibrils is the rule rather than the exception, much insight can be gained from the study of coarse-grained models that capture the key generic features associated with amyloid formation. Here we present a simple lattice model that can capture both protein folding and beta strand formation. Unlike standard lattice models, this model explicitly incorporates the formation of hydrogen bonds and the directionality of side chains. The simplicity of our model makes it computationally feasible to investigate the interplay between folding, amorphous aggregation and fibril formation, and maintains the capability of classic lattice models to simulate protein folding with high specificity. In our model, the folded proteins contain structures that resemble naturally occurring beta-sheets, with alternating polar and hydrophobic amino acids. Moreover, fibrils with intermolecular cross-beta strand conformations can be formed spontaneously out of multiple short hydrophobic peptide sequences. Both the formation of hydrogen bonds in folded structures and in fibrils is strongly dependent on the amino acid sequence, indicating that hydrogen-bonding interactions alone are not strong enough to initiate the formation of beta sheets. This result agrees with experimental observations that beta sheet and amyloid formation is strongly sequence dependent, with hydrophobic sequences being more prone to form such structures. Our model should open the way to a systematic study of the interplay between the factors that lead to amyloid formation.

## Introduction

The ability of many peptides and proteins to convert from their monomeric native state to amyloid fibrils can be attributed to the general ability of polypeptide chains to form intermolecular beta-strands [1]. Simulations, using detailed models, have revealed possible pathways for fibril formation by small peptides [2]–[9]. These models provide valuable information about the factors that determine the free-energy barriers for the nucleation step in the fibril formation pathway and have led to interesting hypotheses about the origins of amyloid toxicity [6], [10], [11]. However, as these models are computationally very expensive, these studies have mostly concerned short segments of amyloidogenic proteins. Yet, to model biologically relevant behaviour of the nucleation pathway, including the formation of oligomers in the initial stages of aggregation, simulation of a substantial number of complete protein chains is necessary; regions of the protein that are not directly implicated in beta strand formation of the amyloid fibrils may still be highly relevant for the aggregation and amyloid formation pathway. In fact, there is evidence that flanking regions can have a considerable effect on aggregation mechanisms [12], [13].

Lattice models have previously been used successfully to model the transition from an unfolded to a folded protein [14] and to model the rearrangement of hydrophobic and polar residues in oligomeric structures [13]. The success of such highly simplified lattice models may at least partly be explained by recent simulations that show that the free-energy landscapes for protein folding in a lattice model are strikingly similar to those obtained for a fully atomistic model [14], [15]. A limitation of existing lattice models is, however, that they do not account for backbone-specific interactions, yet such interactions are vital to understanding the transition from oligomers to fibrils. Some off-lattice coarse-grained models can capture folding specificity [4], [16]. Other off-lattice models can simulate the transition from monomeric peptides to non-specific aggregates [2], [6], [8], [9]. Yet, such off-lattice models, even when coarse grained, are generally too expensive to study the unfolding and rearrangement of multiple proteins.

In this paper, we propose a lattice model that allows both for folding into specific structures and the formation of backbone hydrogen bonds in patterns that are typically observed in amyloid fibres. The model is sufficiently simple to allow extensive simulations of large systems under varying conditions; it also includes explicit directional information of the side chains, and explicit formation of backbone hydrogen bonds between residues. These features make it possible to model the geometric properties commonly observed in beta strands at low computational cost.

In the model developed here backbone residues can make hydrogen bonds with the corresponding residues in neighbouring strands. In addition, the use of an information-based pairwise interaction between the twenty different amino acids, together with the directionality of the side chains, allow for the formation of highly specific structures. As we will show below, beta strands will only form under specific conditions, and when the amino acid composition of the sequence involved creates a favourable environment. Moreover, a previously developed interaction matrix, allows for the simulation of multiple protein molecules without creating unrealistic aggregation behaviour [17].

In what follows, we show that our model allows the design of amino acid sequences that fold into specific target structures. This design process naturally leads to the formation of beta sheets with a hydrophobic and a polar face containing alternating charges. In addition to describing native states, the model can also account for intermolecular beta strands arranged in a cross beta structure, as observed in amyloid fibrils. We find that the formation of such structures is sensitive to the amino acid composition of the peptides. The model proposed here is simple enough to simulate the collective rearrangement of multiple full-length proteins in the initially formed oligomers and the mature amyloid fibrils. The ability of the model to simulate interplay between aggregation and folding is shown in Ref. [18].

## Results and Discussion

Due to the coarse grained nature of the cubic lattice, it is necessary to design sequences that can fold into specific and stable structures. The design process takes a structure as an input, and designs an appropriate sequence. The amino acid composition is altered during the design process, while the structure is kept rigid. Designing a sequence that folds with high specificity can be achieved through minimising the total interaction energy *E* of the sequence based on the input structure. Here we use an adapted version of the minimisation procedure used by Coluzza et al. [14]. In this work the direction of the side chains are also altered in the design procedure and the amino acid distribution is constrained to those of naturally occurring proteins (see methods for further details).

**Figure 1:**
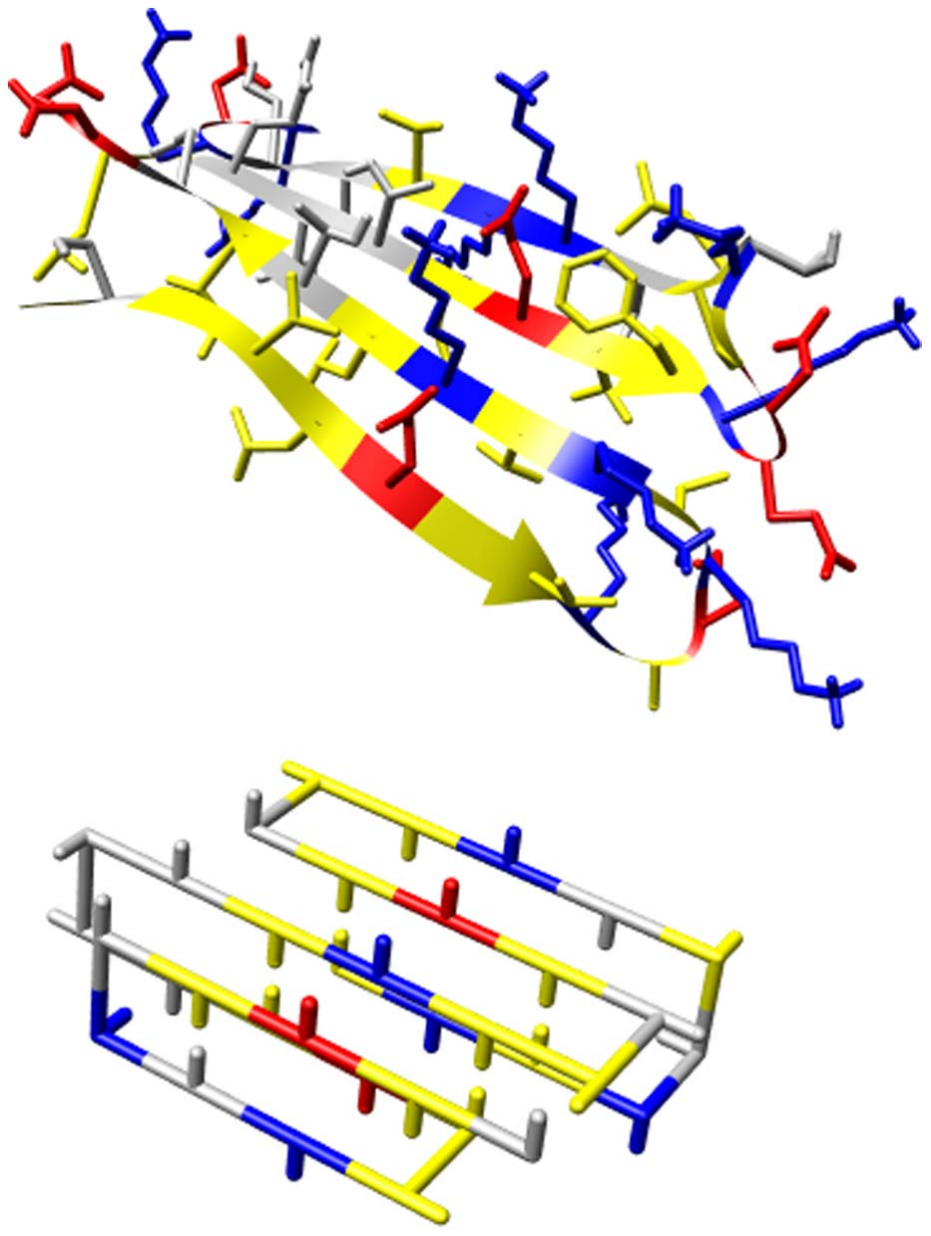
Example of experimental beta sheet structure compared to an on-lattice model. Top: example of a beta sheet in an all-atom structure (1OSP). Note that for clarity only the top sheet of the structure is shown; the hydrophobic downward pointing residues are buried through an another beta sheet. Bottom: example of a designed protein that can fold into its native structure (shown) containing a beta sheet. Note that the designed structure was not explicitly modelled to resemble the experimental structure, but is the result of a stochastic design algorithm (see Methods). Yellow, grey, red and blue residues indicate hydrophobic, polar, negative and positive amino acids respectively.

A typical sequence design for a predefined structure is shown in Figure 1. Just as in the experimental structures (e.g. 10SP), it can be observed that the designed structure contains a hydrophobic core (yellow residues pointing inwards) and a hydrophilic surface (red, blue and grey residues pointing outwards). Moreover, the outer surface of the beta sheet shows alternating positive (blue) and negative (red) residues, similar to the residues in the experimental structure. The realistic amino acid sequence composition of the beta strands demonstrates the biological and physical relevance of the directional amino-acid interaction potential defined by the model.

### Folding with high specificity

**Figure 2:**
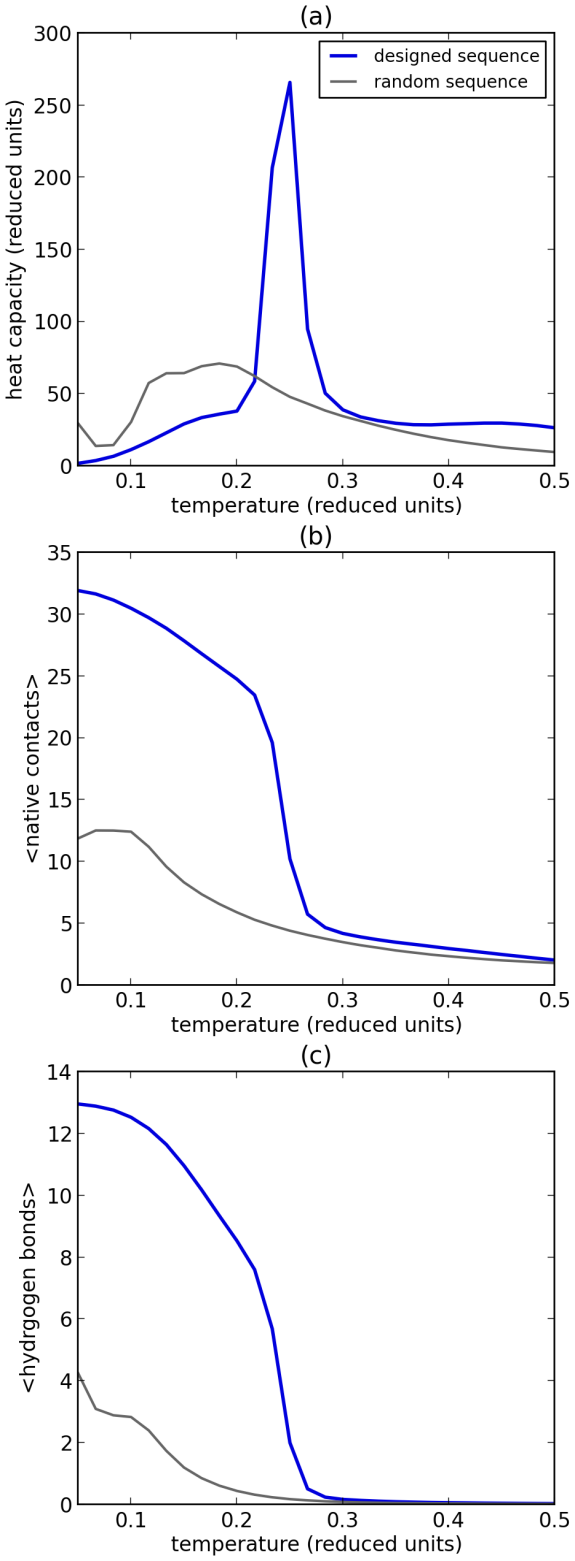
Folding chacteristics and specificity. Folding characteristics are shown for a protein sequence that is designed to fold in a specific structure, and a random protein sequence; both sequences contain 35 residues and have a similar amino acid composition (see Methods). (a) Heat capacity versus temperature. A peak in the heat capacity curve can be observed at the folding transition. (b) Number of native contacts versus temperature. (c) Number of hydrogen bonds versus temperature. From the statistics it is clear that the sequence designed to fold shows a much sharper transitions than a random sequence of the same length. Moreover, the number of hydrogen bonds formed is strongly dependent on the sequence. Please refer to the Methods and Supplement for the sequences and structures used.

Once a sequence has been designed, a Monte Carlo simulation can be used to investigate the folding characteristics of the sequence. We find that the heat capacity of the designed model proteins exhibit a sharp peak in the vicinity of the folding temperature (e.g., Figure 2). Experimentally, such sharp peaks are also observed as a result of folding specificity [19]–[21].

In addition, the folding transition may be detected by considering the average number of native contacts in each configuration of the model protein during the simulation. Native contacts are those contacts that are also present in the structure for which the sequence was designed (the native state). Figure 2 (b) shows that the designed sequences fold well at low temperatures with a high number of native contacts. A sharp transition in this order parameter can be observed from the folded to the unfolded state, at the same temperature as the peak in the heat capacity, indicating again the high specificity of folding. For random sequences with a similar amino acid composition there is no evidence for a sharp ‘folding transition’ in the heat capacity nor in the native contact curves (Figure 2). This result is in agreement with the experimental observation that most random peptide sequences do not fold into well-defined structures [22]. The more gradual transitions for the random sequences may be considered as a collapse into a more compact, molten globule-like state, without a preference for one specific structural arrangement.

Hence at low temperatures, the random sequence forms an ensemble of compact structures, where as the designed sequence folds almost perfectly into its designed structure with high specificity. Similar results have, of course, been obtained by the classic lattice model; however, it is non-trivial (and encouraging) that this feature of specific folding survives in our model where interactions depend on the direction of the side chains. This model is approximately 2–3 fold slower than the classic cubic lattice model as in ref. [17]; typical folding times are around 1–5 CPU hours on a single processor of 2.2 GHz, depending on the length of the sequence.

Lastly, we consider the effect of the hydrogen bonds on the folding behaviour. Figure 2 shows that the ensemble average of the number of hydrogen bonds follows a similar sharp transition to the number of native contacts. This implies that these hydrogen bonds, and therefore the beta strands, cannot be formed unless the side chain interactions are also favourable - as is the case in the folded structure.

Figure 2 also shows that random sequences hardly form any hydrogen bonds. This finding indicates that the interaction potential of the hydrogen bonds is not unrealistically strong and that the formation and stability of beta strands depends on the sequence.

Here a note of caution should also be given: the possibility to form a helical structure is not included in this model; since the stability of beta strands with respect to the disordered coil state is highly sequence dependent, it would not be realistic to model sequences with a high helical propensity on the cubic lattice.

The property that the formation of backbone hydrogen bonds is strongly sequence dependent is in agreement with experimental results: hydrogen bonds between the backbone atoms only form when the side chain interactions are favourable, e.g. refs. [23]–[25].

### Formation of cross-beta fibrillar structures

**Figure 3:**
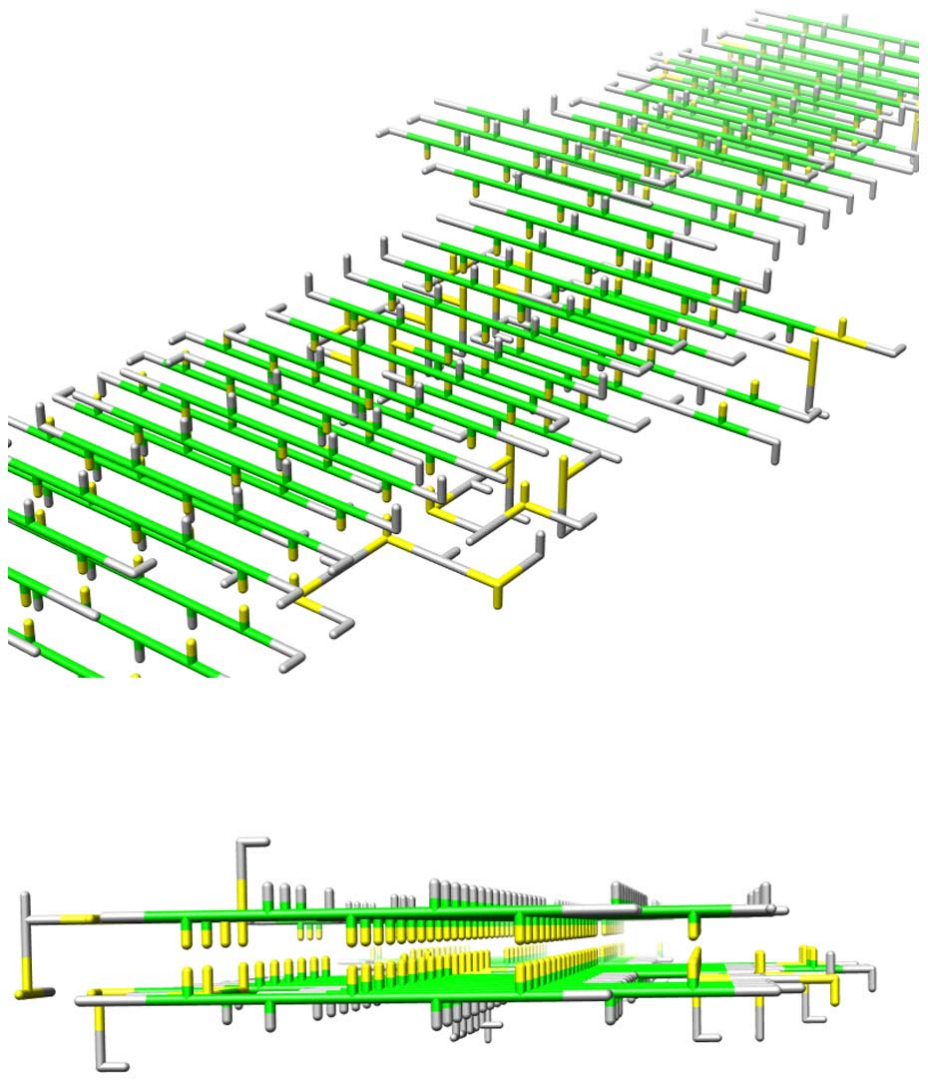
Fibrils with a cross-beta architecture. Top and side view of fibrils formed by a grand canonical simulation with a starting configuration of s small fibrillar structure. The peptides have an alternating hydrophobic (yellow) and hydrophilic (grey) sequence composition. The *strand* and *coil* states are indicated by green and grey respectively.

We simulated several small peptides with an alternating hydrophobic and hydrophilic sequence composition to test the ability of our model to form intermolecular beta sheets. The simulations started from configurations where there were no initial contacts between the different peptides. On simulation both disordered oligomers (i.e. amorphous aggregates) and short fibrillar structures were observed. The short fibrillar structures appeared in relatively long (20 CPU hours), but unbiased, simulations at low temperatures (*T*<0.12) with a constant number of peptides. When the short fibrils are subsequently simulated in a grand canonical ensemble, further growth of the structure may be observed. Figure 3 shows a typical snapshot of such a simulation procedure: long linear fibrillar structures have been formed with a cross beta-architecture.

**Figure 4:**
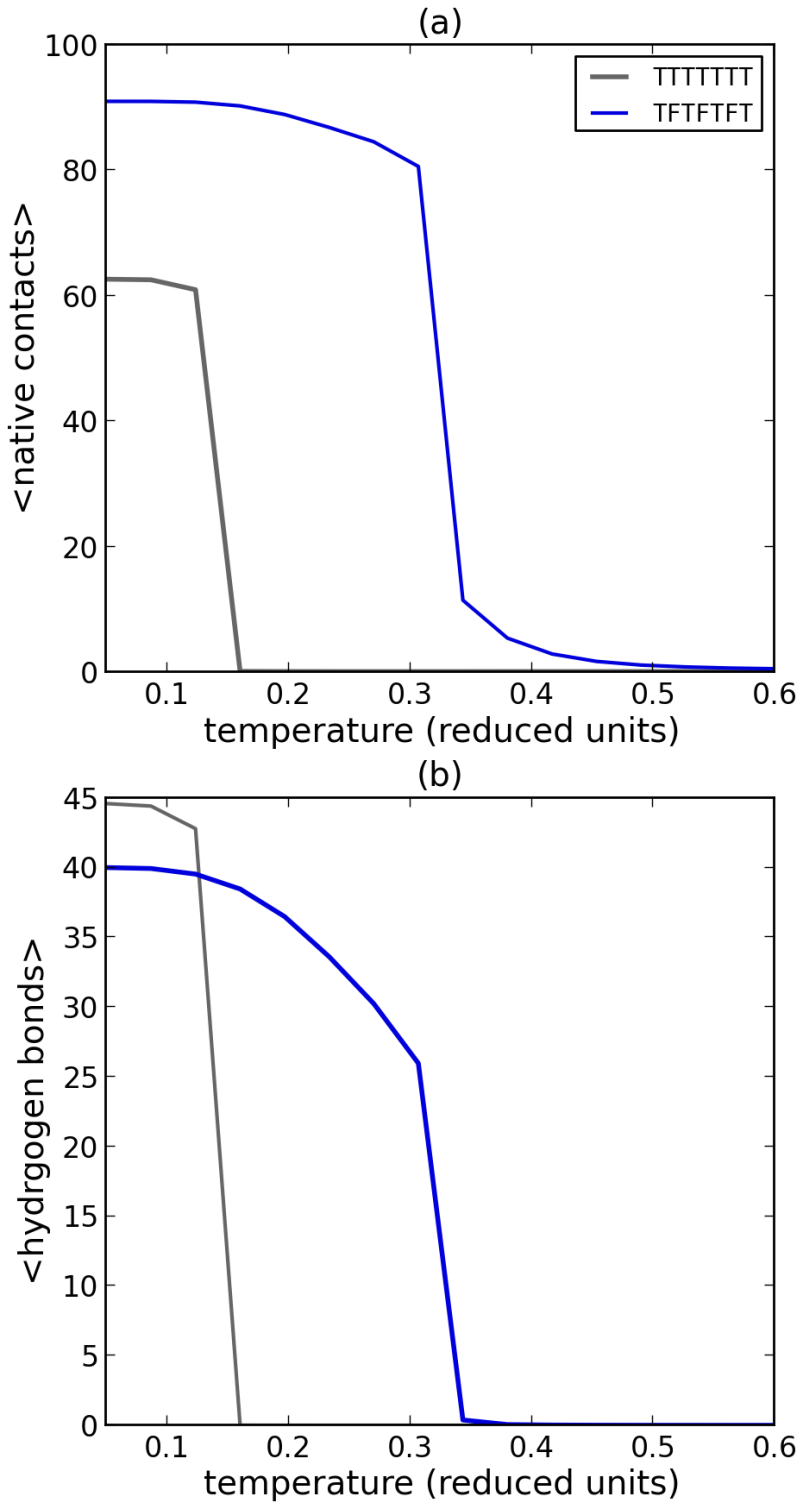
Stability of small fibrillar structures containing 10 peptides. (a) The ensemble average of external (intermolecular) contacts versus temperature for different peptide sequences. External contacts are contacts between different peptides. (b) The ensemble average of hydrogen bonds versus temperature for different peptide sequences. These simulations started from small fibrillar configuration containing 10 peptides with 7 residues and different sequence compositions (see legend). In the temperature regime relevant for folding (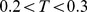), only fibrils that could form a strong hydrophobic core (TFTFTFT) are stable, in this case the hydrophobic residues would point inwards, as shown in Figure 3.

We find that the small fibrillar structures that form at low temperature simulations are extremely stable. We investigated at which temperature such fibrils would become soluble through a series of simulations starting from the fibrillar structure at various temperatures; the initial oligomeric configurations contained 10 peptides, with different sequence compositions. Figure 4 shows at which temperatures the fibrils dissociate; here a high number of intermolecular contacts indicates that the fibrils remain stable (low temperatures) and a low number of intermolecular contacts indicates the peptides are stable as monomers. Hence, these small fibrillar structures, that were initially formed at low temperatures, remain stable at higher temperatures. In addition, such fibrillar seeds enable further growth of the fibrils in grand canonical simulations (Figure 3). Both results are in agreement with a templating (or seeding) mechanism for fibril formation, through which the typical lag times observed in fibril formation may be explained [1], [25], [26].

Figure 4 also shows that the formation of hydrogen bonds occurs at slightly lower temperatures than those at which the first intermolecular contacts form. This suggests that the formation of intermolecular hydrophobic interactions is stronger and enables subsequent hydrogen bonding between peptides. Note that similar observations have been made by all-atom modelling [27], [28]. Comparing the peptide sequences with different amino acid compositions shows that hydrogen bond formation is strongly sequence dependent. Hence in this model both favourable interactions between side chains and hydrogen bonding are necessary for the creation of beta strands in the fibrils. This is particularly evident for the temperature range relevant for protein folding (02.<*T*<0.3).

## Conclusions

In this work we have presented a very simple protein lattice model that includes directional information of the side chains and ability to form hydrogen bonds. Our results show that this model can be used to design sequences that fold with high specificity into predefined structures. Moreover, simulations with the model show that hydrogen bonds are formed both in beta sheet motifs in folded proteins, and in intermolecular cross-beta structures in fibrils formed from small peptides. Most importantly, the simplicity of the model makes simulations feasible that investigate the interplay between folding, fibril formation and amorphous aggregation [18]. The full model, given as source code, is available at http://www.few.vu.nl/~abeln/hb-lattice.

## Methods

### The model

As a basis for the model presented here, we use the classic cubic lattice model as described in [13], [14], [17], [29], [30]. In this classic lattice model each residue is located on a point of the cubic lattice and is assigned one of twenty amino acid types. In the remainder of this section, only the differences between the model developed here and the model described in refs. [13], [17] will be discussed.

In our model, each residue has a side chain direction, 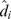, and a state representing the secondary structure, 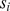. In a structure of N residues, we now have for each residue 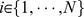:
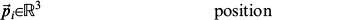


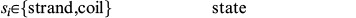


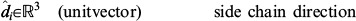


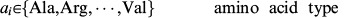



Note that all residue positions 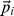 are situated on the cubic lattice. The side chain directions 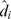 do not occupy any colume and point from their residue's position to a neighbouring lattice site. The side chain is not allowed to point in the same direction as the backbone, leaving a choice of four possible directions for each side chain; the side chains situated at the end of the protein chain, have a choice of five directions.

A residue makes a contact with another residue when it is situated on a neighbouring lattice point, and is not a sequential neighbour in the chain. A contact 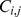 between two residues *i* and *j* is thus defined as:
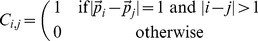
(1)


#### Potential energy

The total energy of the system can be split into several different and independent components including the hydrogen bond energy (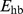), the interaction energy between amino acids (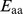), the energy of the states (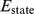) and the interaction energy of each amino acid with the solvent (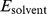):
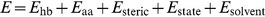
(2)


Here the contributing potential energy terms are functions of the following variables:
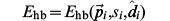


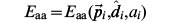


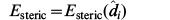


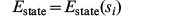


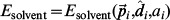



Note that in the model described here no explicit energy is attributed to the state of the residue, i.e. 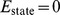. Instead, favourable hydrogen bonding interactions will bias residues towards the appropriate state.

#### Hydrogen bonds

**Figure 5:**
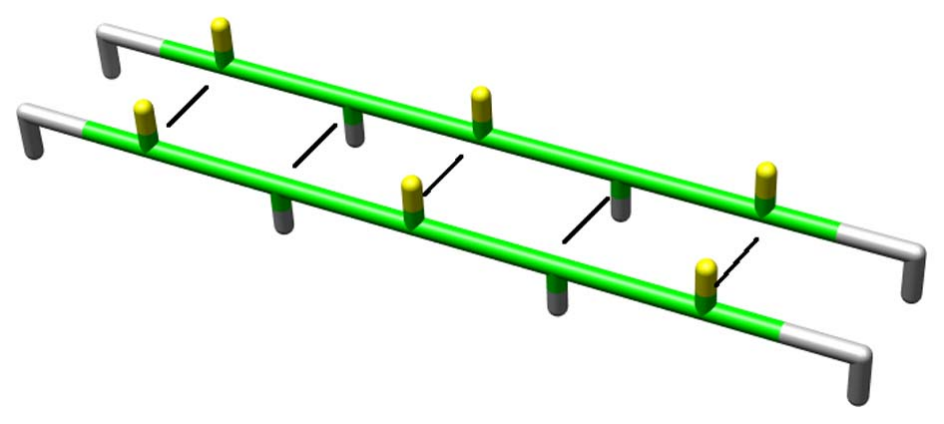
Hydrogen bonds formed between two strands. The black lines represent hydrogen bonds; residues in green are in the *strand* state. Hydrogen bonds are allowed to form when neighbouring residues are in a *strand* state and the side chains are oriented in the same parallel direction.

The total potential energy of the hydrogen bonds for a configuration is given by:
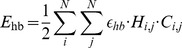
(3)


where 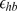 represents the potential energy per hydrogen bond and 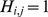 indicates whether or not a hydrogen bond between residues *i* and *j* exists (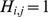 in case of a hydrogen bond). Hydrogen bonds are only allowed between two residues that are both in the ‘strand’ state, and when their side chains are oriented in the same direction (Figure 5), thus:
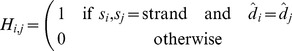
(4)


#### Interactions between amino acids

**Figure 6:**
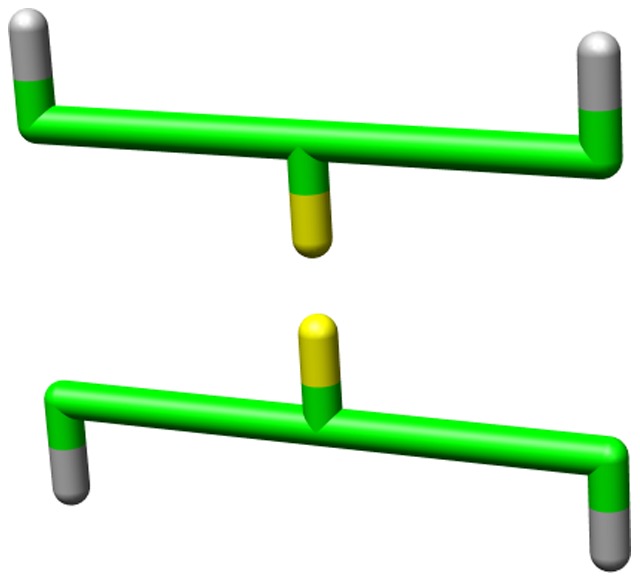
Facing side chains that interact. The yellow residues interact due to their orientation: they are directed towards each other.

**Figure 7:**
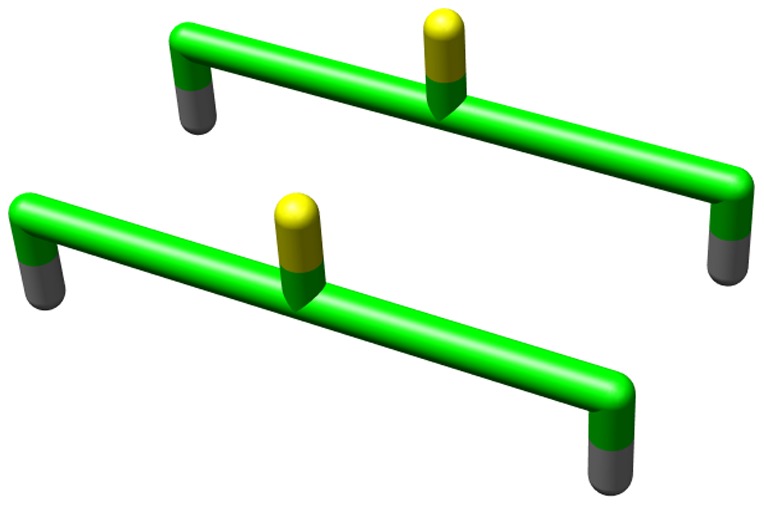
Parallel side chains that interact. The yellow residues interact, since they point in the same direction in a parallel fashion.

In the presented model the total potential energy for pairwise interactions between amino acids depends both on the positions on the lattice and side chain directions of the two residues. The potential energy of pairwise amino acid interactions, 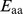, is given by:
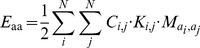
(5)


where 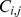 indicates whether or not the residues are in contact, as before, and 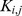 indicates whether the directions of residues *i* and *j* allow interation. Elements of the interaction matrix *M* provide the strengths of the pairwise interaction energies between different types of amino acids (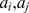). The directions of the side chains are allowed to interact when the side chains of the residues face each other (Figure 6) or when the side chains lie parallel to each other, while being oriented in the same direction (Figure 7), thus:
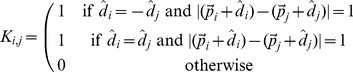
(6)


#### Steric hindrance penalty

To prevent consecutive side chains being oriented in the same direction, a steric hindrance energy term is used. In real protein structures such conformations are blocked as a result of steric hindrance from clashes between side chain and backbone atoms; such detail is not included in this model. To prevent these conformations being present, we use:
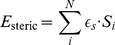
(7)


Here 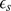 is the energy penalty for steric hindrance and *S_i_* indicates whether or not residue *i* is in a state that causes steric hindrance:
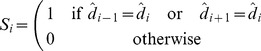
(8)


#### Solvent interactions

Interactions between the solvent, mimicked by vacant lattice sites, and a given residue depend on the particular amino acid type, the direction of the side chain and the position of the residues with respect to the solvent. The total solvation energy, 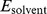 is given by:
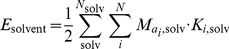
(9)


Here 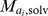 is the column of the interaction matrix that gives the interaction strength between the solvent and residue type 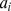 (see below); 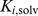 indicates whether or not an interaction between the solvent and residue *i* occurs. The residue must in contact with a solvent site and its side chain directed towards the solvent (empty lattice site), thus:
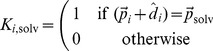
(10)


#### Parameter values

Note that for real proteins the precise contributions towards the stability of a protein for backbone hydrogen bonding compared to side chain interaction energies is still a topic of discussion. Nevertheless, the side chain interactions, including hydrophobic effects, appear to be the dominant forces behind protein folding [31]. The current parametrisation of this model, as given in Table 1, is in agreement with this statement.

**Table 1:** List of parameter values for the model.

parameter	value	
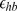	−50	hydrogen bonding energy
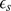	55	steric hindrance energy
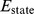	0	strand/coil energy
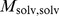	0	solvent self interaction

#### Interaction matrix

The pairwise interaction strengths (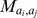) between amino acids and between amino acids and the solvent are defined as in ref. [17]. The explicit row of interactions between the solvent and amino acids can be rationalised in the current model - as side chains can explicitly orient towards the solvent. In addition this potential has been shown to prevent unrealistic aggregation behaviour for proteins in their native states.

### Monte Carlo simulation

#### Classic Monte Carlo algorithm

To simulate configurations of the model presented here, we use a Monte Carlo simulation algorithm. Trial steps are accepted according to:
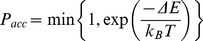
(11)


where 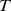 is the simulation temperature, 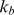 is the Boltzmann constant and 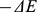 is the difference in energy between the new and old configuration of the system. Trial moves are either internal moves, changing the configuration of a chain (end move, corner flip, crank shaft, point rotation), or rigid body moves, changing the position of the chain relative to other objects (rotation, translation), see ref. [13], [14] for more details. In addition, local moves to change the states and side chain directions are performed (see below).

At each iteration a single local trial move and a global trial move (including point rotations) with the probability 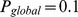 are performed.

#### Moves between strand and coil states

The state of each residue may be altered by a local move from *strand* to *coil*, and vice versa. The transition from *coil* to *strand* is only allowed when the following criteria are satisfied:

1. There is no turn in the backbone at residue 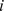

2. Side chains of sequential neighbour are oriented into the opposite direction, if the neighbouring residues are in the *strand* state

Note that the potential energy of hydrogen bonds is taken into account when making the Monte Carlo move to change the state of a residue, since any move is accepted according to the criterion defined in Eqn. 11. Residues in the *strand* state are not permitted to change their backbone configuration or their side chain direction. Hence the *strand* state will be entropically unfavourable; this may, however, during the simulation be compensated by an enthalpic contribution from hydrogen bonding.

#### Moves of side chain direction

The side chain direction is altered during the simulation with local moves. In such a move, a random new direction is chosen for the side chain, provided that it does not overlap with the direction of the backbone. Each move is accepted or rejected according to the criterion in eqn. 11.

#### Simulation setup

The volume of the simulation box was kept constant at 80 × 80 × 80 lattice points for the folding simulations and at 30 × 30 × 30 lattice points for the small fibril structure simulations.

Parallel tempering, or temperature replica exchange, is used to converge more rapidly to sampling of equilibrium configurations for the folding simulations. Multiple simulations at different temperatures are run in parallel, while attempting to swap temperatures every 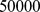 moves with 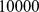 trial temperature swaps in each simulation. A trial swap between the temperatures of two replicas is accepted with a probability [32]–[34]:
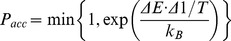
(12)


A grand canonical Monte Carlo simulation is performed to investigate the growth of the fibril seeds at a constant (low) osmotic pressure, see ref. [17] for further details.

### Sequence design

One of the challenges when using a cubic lattice model is to design a sequence that will fold into a predefined structure. Previously, it has been shown that one can obtain good folding sequences by minimising the potential energy of the folded state, while keeping the sequence variance high [14]. Here we follow a similar approach, but use a different function to determine the sequence variance.

We can keep the sequence variability high by keeping the distribution of amino acid types close to that observed in nature; here we use the same set of experimental protein structures as used in Ref. [17] to obtain this distribution. First we define a distance 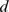 between the distribution of amino acids types in the experimental set and the distribution in the sequence that is to be designed:
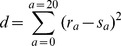
(13)


Here 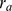 denotes the fraction of amino acids with type 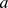 in reference to the total number in the experimental set; 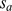 denotes the fraction of amino acids with type 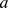 in the sequence that is to be designed. The sum is over all 20 types of amino acids. The distance defined above needs to be kept small, to design a sequence with a wide variety of amino acids.

A suitable acceptance criterion is given by:
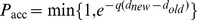
(14)


where 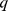 is a constant that sets the strength of the biasing potential. This acceptance rule is used in addition to the acceptance rule for the potential energy of the sequence, as in ref. [14].

If we change amino acid 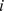 for amino acid 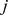, then 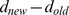 simply becomes.
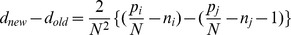
(15)


where 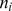 and 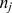 are the number of amino acids of type 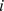 and 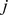, respectively, before the change.

Note that this approach is as effective in designing folding sequences, as the previously described variance rule, but it gives sequence compositions that are closer to those observed in nature.

### Sequences and structures

To generate the results in Figure 2 a designed sequence, see procedure above, and a random sequence of 35 residues with a similar amino acid content were simulated (the designed sequence reads TLSINDYGESEPFKVAVCELQNDDIHIKSLRPARCG and the random sequence PEAMIGPLTGAIHFKVSTSNWGREDLEDVYRQANLI).

For Figure 4 ten peptides consisting of seven residues were used with two different sequences (TTTTTTT and TFTFTFT); the simulations were started from a fibrilar configuration that was formed by simulating ten TFTFTFT peptides with long simulations at a low temperature. The sequences and structures used in this work may be found as PDB files at http://www.few.vu.nl/~abeln/hb-lattice.
